# Correlation of blood pressure and the ratio of S1 to S2 as measured by esophageal stethoscope and wireless bluetooth transmission

**DOI:** 10.12669/pjms.294.3639

**Published:** 2013

**Authors:** Kyoung Hoon Lim, Young Duck Shin, Sang Hi Park, Jin Ho Bae, Hong Jae Lee, Seon Jung Kim, Ji Yun Shin, Young Jin Choi

**Affiliations:** 1 Kyoung Hoon Lim, Department of Anesthesiology and Pain Medicine, Chungbuk National University Hospital, Republic of Korea.; 2 Young Duck Shin, Department of Anesthesiology and Pain Medicine, Chungbuk National University Hospital, Republic of Korea.; 3 Sang Hi Park, Department of Anesthesiology and Pain Medicine, Chungbuk National University Hospital, Republic of Korea.; 4 Jin Ho Bae, Department of Anesthesiology and Pain Medicine, Chungbuk National University Hospital, Republic of Korea.; 5 Hong Jae Lee, Department of Anesthesiology and Pain Medicine, Chungbuk National University Hospital, Republic of Korea.; 6 Seon Jung Kim, Department of Anesthesiology and Pain Medicine, Hankook General Hospital, Republic of Korea.; 7 Ji Yun Shin, Department of Biomedical Engineering, Chungbuk National University Hospital, Republic of Korea.; 8 Young Jin Choi, Department of Surgery, Eulji University Hospital, Republic of Korea.

**Keywords:** Blood pressure, Esophageal, Heart sounds, Stethoscope

## Abstract

***Objective*** *:* Esophageal stethoscope has the advantage of being non-invasive, easily placed and capability to monitor the heart sound. This study was designed to determine whether the ratio of S1 to S2 analyzed by esophageal stethoscope and wireless bluetooth transmission can be accurate indicator that express the correlation with blood pressure.

***Methods:*** Total 33 adult male and female without cardiac disorder and with normal heart rhythm were selected randomly as the subjects of this Study. Two microphones were used with one for acquisition of heart sound by connecting it to the esophageal stethoscope while the other was used to measure the background noise in the operating room. After having transmitted the heart sound measured with the esophageal stethoscope to the receiver by using bluetooth module, it was saved in PC and outputted, following removal of noise in the operating room and the respiratory sound. S1 and S2 were measured with computation of the ratio of S1 to S2. Correlations between the systolic blood pressure with each of the S1, S2 and ratio of S1 to S2 were examined by using correlation analysis.

***Results:*** The ratio of S1 to S2 displayed the highest correlation with the systolic blood pressure, with S1 and S2 also displaying positive correlation with the systolic blood pressure.

***Conclusion:*** As the result of analysis of the heart sound and the systolic blood pressure measured by using the esophageal stethoscope, the radio of S1 to S2 displayed greater correlation with the systolic blood pressure in comparison to the S1.

## INTRODUCTION

Among the monitoring activities on surgery patient, the monitoring of cardiac function is highly important, and the method of such monitoring includes measurement of blood pressure and heart beat, ECG, echocardiography and heart sound hearing, etc. Among these monitoring methods, the esophageal stethoscope has the advantage of being non-invasive, ease of placement, and being able to measure not only the heart sound but also to monitor the temperature and the respiratory sound of the patients. The measurement of heart sound through the esophageal stethoscope is more useful in measuring the changes in the heart sound consistently in accordance with time in comparison to the precordial stethoscope since it is fixed at one location throughout the surgery. In the case of a specially designed stethoscope, ECG through esophagus can be recorded, which can be useful in diagnosis of atrial arrhythmia and right or left ventricular posterior wall ischemia. In addition, the esophageal stethoscope is effective in the treatment of sinus bradycardia or atrio-ventricular rhythm.^1^

Although numerous researches that reported correlations between hemodynamic indices such as the ejection fraction and the ratio of S1 to S2 in previous researches using the precordial stethoscope^2^ were carried out, there are not many researches that analyzed the heart sound and on its relationship with the hemodynamic indices using the esophageal stethoscope. Accordingly, this study aims to analyze the heart sound by using the esophageal stethoscope and the wireless bluetooth transmission, and to examine whether the ratio of S1 to S2 can be used as objective index with correlation with the blood pressure.

## METHODS

Thirty three (33) adult male and female without cardiac disorder and with normal heart rhythm who corresponded to the physical class classification I or II of the American Society of Anesthesia (ASA) among the patients for whom general anesthesia was planned were selected randomly as the subjects of this study (Table-I). This study was carried out by obtaining the approval of Institutional Review Board and consents of the patients after having sufficiently explained the purpose and method of the study in advance.

**Table-I:** Demographic Data (N = 33

Age (yr)	46.8 ± 13.5
Weight (kg)	67.7 ± 10.7
Height (cm)	164.1 ± 9.7
Sex (M/F)	18/15
ASA (I/II)	23/10

Values are means ± SDs

**Table-II:** Correlation between Blood Pressure and Sound Pressure Level.

		S1/S2	smpl^†^(S1)	smpl(S2)
SBP^*^	Pearson coefficients	0.914	0.785	0.373
	P value	< 0.001	< 0.001	0.033

^*^Systolic blood pressure, ^†^Sound milli pressure level

**Fig.1:**
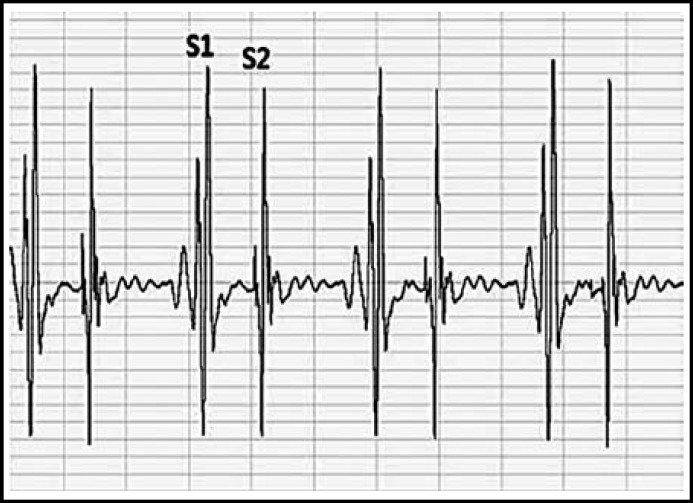
The Amplitude Waves of Heart Sounds Recorded by Esophageal Stethoscope. S1: first heart sound, S2: second heart sound

**Fig.2:**
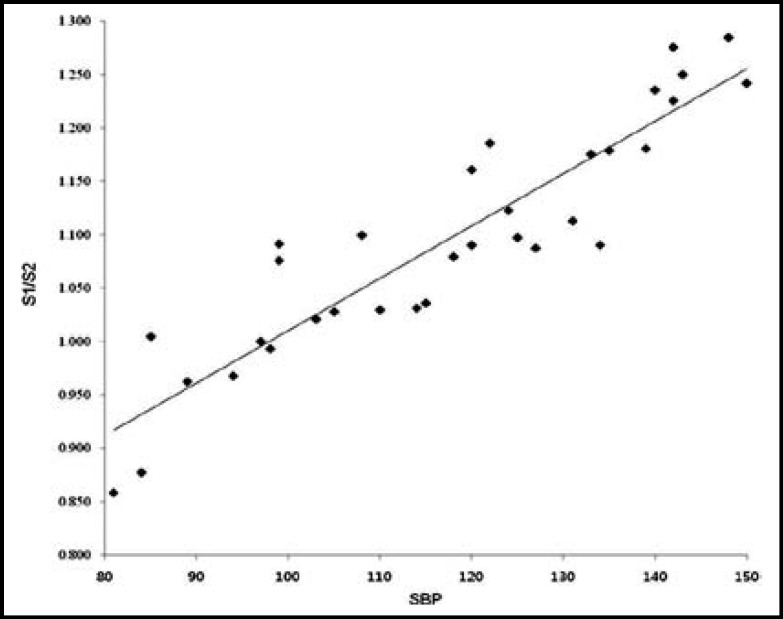
The scatter plot shows the highest positive correlation between SBP and S1/S2. S1: first heart sound, S2: second heart sound, SBP: systolic blood pressure

**Fig.3:**
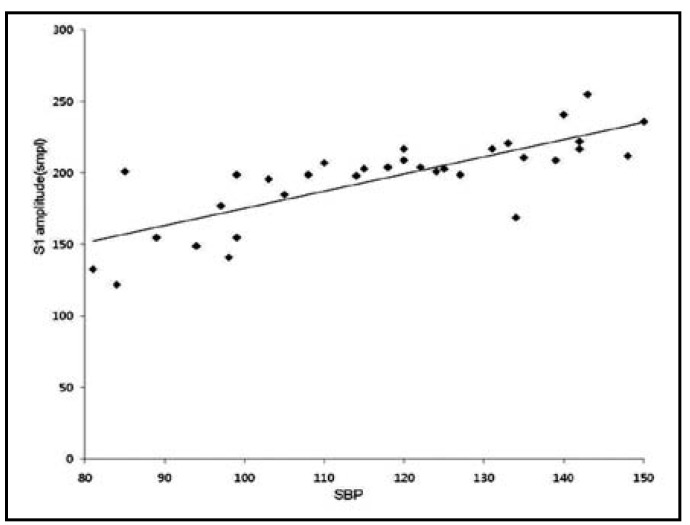
The scatter plot shows the positive correlation between SBP and S1. S1: first heart sound, SBP: systolic blood pressure, smpl: sound milli pressure level

**Fig.4:**
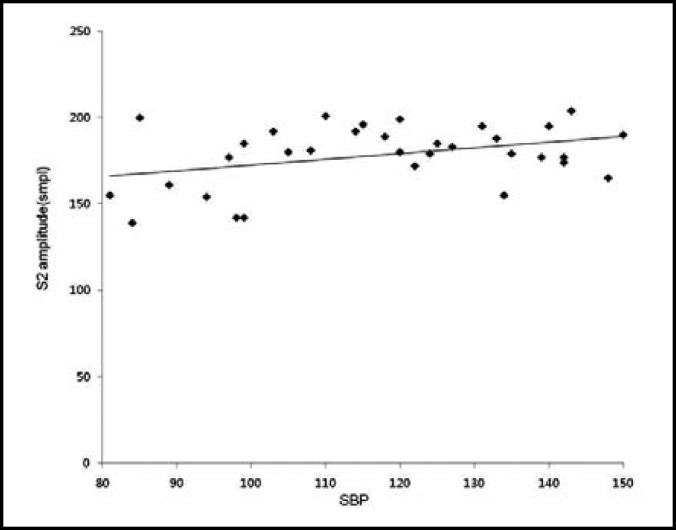
The scatter plot shows the lowest positive correlation between SBP and S2. S2: second heart sound, SBP: systolic blood pressure, smpl: sound milli pressure level

All the patients arrived at the operating room without undergoing premedication, and total intravenous anesthesia was induced with propofol and remifentanil by using the target controlled infusor (Orchestra^®^, Fresenius Vial, France). The radial artery was secured for continuous measurement of the blood pressure and the esophageal stethoscope was positioned to listen to heart sound. The esophageal stethoscope was positioned at the depth of 28-32 cm from upper incisor where the S1 sound was heard the loudest, the location reported to be the best position to listen to heart sound^3^, and the omnidirectional microphone with frequency response range of 20-20,000 Hz (MKE 2-5-3 goldC; Sennheiser, Germany) was connected.

Two microphones were used with one for acquisition of the hearth sound by connecting it to catheter while the other for measurement of the noise in the operating room. ATmega128L (ATMEL, USA) for application in 8-bit control was used as micro-controller, while AD822 (Analog Device Inc, USA) was used as the amplifier and FB570 (Firmtech, Korea) as the bluethooth module. Receiver plays the role of storing in the PC and outputting the heart sound data transmitted from the transmitter after having received and signal processed the data. Portion of the recorded data through the esophageal stethoscope that coincided with the noise in the operating room was removed and filtering was performed by using Cool Edit Pro 2.1(Syntrillium Software Corporation, Phoenix, USA) and noise such as respiratory sound, etc was removed with low-pass filtering.

Since the intensity of heart sound can differ according to breathing, comparison was made consistently with the end-tidal blood pressure. S1 and S2 were measured at 5 minutes after induction of anesthesia. They were measured as the mean value of data for 3 seconds, data with the radio of S1 to S2 computed separately. The sound pressure was indicated with the sound pressure level (SPL) and expressed with the unit of sound milli pressure level (SMPL). Correlation with each of S1, S2 and the radio of S1 to S2 was analyzed on the basis of the systolic arterial blood pressure. 

SPSS (version 12.0, SPSS Inc, Chicago, USA) was used for statistical analysis. Pearson correlation was used for analysis of correlation between the blood pressure and each of the heart sounds, and it was determined to be statistically significant if P value is less than 0.05. 

## RESULTS

The heart sound measured through esophageal stethoscope was stored in the receiver through the wireless Bluetooth transmission, and S1 and S2 were easily distinguished by removing the external noise through the computer recording program and filtering (Fig.1). As the result of analysis of correlation between the systolic blood pressure and each of the variables, the ratio of S1 to S2 displayed positive correlation with Pearson’s correlation coefficient of 0.914, which was the highest correlation in comparison to the other variables (Table-II, Fig.2). The systolic blood pressure and S1 displayed positive correlation with Pearson’s correlation coefficient of 0.785 (Table-II, Fig.3) while that with the S2 displayed the lowest positive correlation with Pearson’s correlation coefficient of 0.373 (Table-II, Fig.4).

## DISCUSSION

Chest auscultation^4^ and phonocardiography^5^ have been studied in various researches. Vibration associated with sudden acceleration or deceleration of blood in the cardiovascular system is the key compositional element of the heart sound.^6^ This is divided largely into the 1^st ^heart sound (S1) generated at the time of the closure of the mitral valve and the tricuspid valve, and the 2^nd ^heart sound (S2) generated at the time of closure of the aortic valve and the pulmonary valve. S1 is a slightly low-pitch sound that is heard the loudest at the apical region as the result of the closure of atrioventricular valve, while S2 is a slightly high-pitch sound heard at the apical region as the result of the closure of arterial valve.^7^

S1 is composed of M1 that is generated with the closure of mitral valve and T1 generated with the closure of tricuspid valve, and there is hypothesis that it is related to the movement of blood.^8^ S1 increases in the event of shortening of the diastolic phase due to tachycardia, increase in blood flow from atrium to ventricle due to increase in the cardiac output, increase in blood flow from atrium to ventricle due to the mitral valve stricture, and contraction of ventricle with abnormally short interval following the contraction of atrium similar to shortening of the PR interval.^6^

The mitral valve closes when the left ventricular pressure exceeds the left atrial pressure in the heart cycle, and, since the blood flows from high to low pressure, the mitral valve closes in order to prevent counter flow of blood from the left ventricle to the left atrium as the left ventricular pressure becomes larger. Greater and quicker the increase in the left ventricular pressure, more powerful the closure of mitral valve, thereby increasing the intensity of S1. Therefore, on the basis of this fact, it is possible to presume that S1 is related to the myocardial contractile force. In fact, there is a report that S1 reflects the myocardial contractile force as the result of simultaneous monitoring of echocardiography, Doppler flow and phonocardiogram.^9^^-^^11^

In addition, there is a report in a research using experimental dog that when the systolic left ventricular pressure is increased through injection of inotropics, S1 increases in proportion^12^ as well as report that amplitudes of S1 and S2 decrease following induction of inhalation anesthetics.^13^ S2 is divided into A2 arising from the closure of aortic valve and P2 due to the closure of pulmonary valve, and the intensity of S2 increases with greater intensity in the closure of aortic valve. The aortic valve closes in order to prevent counter flow of blood, which was infused into the aorta from the left ventricle, into the left ventricle again.

The closure of aortic valve is caused by the difference in the aortic pressure and the left ventricular pressure, and the intensity of its closure increases with the increase in the difference in these pressures. Such pressure difference is determined mainly by the internal pressure of the aorta because the left ventricular pressure at diastolic phase under the normal situation is close to zero. Resultantly, the internal pressure of the aorta is the force that closes the aortic valve. If situation in which increase in the flux of blood from the left ventricle to the aorta occurs, the aorta is further expanded and the internal pressure of the aorta increases in proportion, thereby resulting in the increase in the intensity of closure of the aortic valve with ensuing increase in the intensity of S2. There also is a report that intensity of S2 decreases in patients with reduction in the ejection fraction in comparison to normal person.^14^

Under normal situation, S2 is clearly divided into A2 and P2 because of the delay in the closure of pulmonary valve with increase in the stroke volume and the time with increase in the flux of blood into the right ventricle at the time of diastolic phase.^6^ The time of the closure of aortic valve is not affected significantly by breathing. Since the lung plays the role of buffering the changes in the blood flow, blood that flows into the left ventricle through the left atrium from the lung is maintained almost consistently throughout the breathing cycle. In addition, paradoxical splitting in which P2 occurs prior to A2 may occur. The most common reason for this phenomenon is the delay in the excitement of the left ventricle due to left bundle branch block and right ventricular extra-heartbeat.^6^^,^^15^ In this Study, A2 and P2 would not have been clearly distinguished since the blood pressure and the heart sound was measured at the end of the exhalation phase simultaneously.

In this Study, those with cardiac disorders and irregular rhythm were excluded because such could affect the intensity of S1. Since the diastolic phase volume changes if the interval of the heart beat is irregular as in the case of atrial defibrillation, the intensity of S1 can change at every interval due to changes in the contractile force of the left ventricle at every heartbeat. In the case of complete AV block, S1 with enormous sound (cannon sound) can occur with the contraction of both the atrium and the ventricle occurring completely simultaneously at times.^16^^,^^17^ In addition, the intensity of S1 decreases if the increase in the left ventricular pressure heart beat is slow, if the PR interval is long and if the valve is closed incompletely due to the reduction in the valve tissue as in the case of mitral valve closure deficiency.^6^^,^^15^

Although there is not much research result on the relationship between the ratio of S1 to S2 (S1/S2) and the hemodynamic indices, there is a report that S1/S2 is reduced with lowering of the ejection fraction and lowers the changes in the ventricular pressure (dP/dt) for each unit time in research that used echocardiography, catheter and phonocardiogram.^2^ There also is a report that S1/S2 is reduced with reduction in contractile force as the result of analysis of the heart sound while performing percutaneous coronary intervention.^18^ It is possible to establish hypothesis that S1/S2 is related to the myocardial contractile force and correlated to the blood pressure on the basis of these results. While all of S1, S2 and S1/S2 were correlated with the blood pressure, S1/S2 displayed the highest correlation. This signifies that the changes in S1 is greater than the changes in S2 in accordance with the changes in the blood pressure, and it is deemed to be the result of S1 better reflecting the myocardial contractile force than S2. In addition, based on the results, S1/S2 is determined to be more appropriate as an index to indicate the hemodynamic changes in comparison to S1.

Esophageal stethoscope and heart sounds have been studied in various clinical studies. Nezafati MH et al^19^ demonstrated that the intraoperative esophageal stethoscope provides a remarkably effective technique for monitoring and evaluating patent ductus arteriosus (PDA) ligation. Kahrom M et al^20^ used esophageal stethoscope to research cardiac murmurs in PDA. Although the precordial stethoscope can be used to analyze the heart sound, analysis is difficult and complicated due to noises and overlapping of sound. Accordingly, this experiment used the esophageal stethoscope. Noise in the operating room was measured separately and removed using program, and breathing noise could be removed by executing simple low-pass filtering. Although the left ventricular function could be assessed, and counter-flow of valve, stricture, area and pressure difference and the contractile force of ventricle could be measured accurately by using the echocadiography and Doppler for the purpose of the monitoring of the surgery patients,^21^ these equipment are expensive with specialized and complex process of installation and analysis.

Therefore, it would be possible to more easily analyze the heart sound and monitor the hemodynamic changes of the patients by using the esophageal stethoscope and the wireless Bluetooth transmission for which installation and analysis is relatively simple if additional researches on the relationship between the hemodynamic changes and the heart sound are carried out in the future.

## CONCLUSION

As the result of analysis of the heart sound and the systolic blood pressure through the esophageal stethoscope and the wireless Bluetooth transmission, it was possible to confirm that the blood pressure has positive correlation with each of S1, S2 and S1/S2. Since S1/S2 displayed the highest correlation among these, it could be used as an appropriate index to display the correlation with the blood pressure. In addition, development of equipment for the monitoring of the hemodynamic changes of patients by using the esophageal stethoscope and the wireless Bluetooth transmission, for which installation and analysis is simple, could be expected through additional researches. 

## Authors Contribution:

KHL & YDS: Conceived, Designed and did statistical analysis & editing of manuscript.

SHP, JHB, HJL, SJK, JYS & YJC: Did data collection and manuscript writing.

YDS: Did review and final approval of manuscript.
